# Causal Relationship Between Post‐Traumatic Stress Disorder and Immune Cell Traits: A Mendelian Randomization Study

**DOI:** 10.1002/brb3.70073

**Published:** 2024-09-30

**Authors:** Jian Wang, Yuan Shao, Xianhua Deng, Jianbin Du

**Affiliations:** ^1^ Shenzhen Mental Health Center Shenzhen Kangning Hospital Shenzhen Guangdong China; ^2^ Department of Geriatric Psychiatry The Affiliated Mental Health Center of Jiangnan University, Wuxi Central Rehabilitation Hospital Wuxi Jiangsu China

**Keywords:** immune cells, inflammatory, Mendelian randomization study, post‐traumatic stress disorder

## Abstract

**Introduction:**

Post‐traumatic stress disorder (PTSD) is a debilitating psychological disorder that occurs after exposure to catastrophic‐level experiences. Although alterations in immune function have been identified in individuals with PTSD, the causal relationship between the two remains unclear.

**Methods:**

To investigate the causal relationship between PTSD and immune function, we conducted the forward and backward two‐sample Mendelian randomization (MR) analyses, based on summary‐level genome‐wide association studies (GWAS) data on PTSD and immune cell traits.

**Results:**

For the forward MR analysis, PTSD was found to reduce the levels of CD62L− dendritic cell (DC) (beta = −0.254, FDR = 0.01), CD86+ myeloid DC (beta = −0.238, FDR = 0.014), CD62L− myeloid DC (beta = −0.26, FDR = 0.01), CD62L− CD86+ myeloid DC absolute count (beta = −0.264, FDR = 0.024), and CD62L− CD86+ myeloid DC (beta = −0.328, FDR = 0.002). In contrast, PTSD was observed to increase the level of CD28− CD8dim T‐cell absolute count (beta = 0.27, FDR = 0.029). For the backward MR analysis, the odds ratio (OR) for CD33 on CD33dim HLA DR+ CD11b− in relation to PTSD risk was found to be 1.045 (95% CI = 1.021–1.069, FDR = 0.008). The OR for FSC‐A on HLA DR+ CD8br was 1.048 (95% CI = 1.018–1.079, FDR = 0.039) and for CCR2 on CD14− CD16+ monocyte was 1.059 (95% CI = 1.027–1.092, FDR = 0.008). No significant pleiotropy was detected in both forward and backward MR analyses.

**Conclusion:**

The bidirectional MR study shed light on the intricate interplay between immune function and PTSD. The identification of a bidirectional causal relationship between T cells and PTSD opens new avenues for considering innovative approaches to the prevention and early intervention of PTSD.

## Graphical abstract

We explored the causal relationship between post‐traumatic stress disorder (PTSD) and immune cells using a Mendelian randomization study approach on the basis of publicly available genome‐wide association studies (GWAS) data. The results revealed that PTSD affects some immune cell levels, whereas individual immune cells also increase the risk of developing PTSD. This demonstrates the complex relationship between PTSD and immune function.

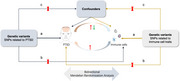

## 1 Introduction

Post‐traumatic stress disorder (PTSD) is a debilitating psychological condition that arises subsequent to exposure to a profoundly distressing or life‐threatening event (Maercker et al. 2022; Hori and Kim 2019). Clinically, individuals afflicted with PTSD commonly exhibit persistent symptoms, encompassing traumatic re‐experiencing, avoidance behaviors, and heightened vigilance, all of which contribute to significant distress and functional impairment (Bisson et al. 2015; Qi, Gevonden, and Shalev 2016). The global lifetime prevalence of PTSD is estimated to be approximately 3.9% (Koenen et al. 2017), rendering it a matter of considerable public health concern. In pursuit of the overarching goal of early prevention and intervention for PTSD, the pivotal focus of current research lies in elucidating the factors that influence its development and its underlying pathophysiology.

An expanding body of research has revealed a robust correlation between PTSD and the immune system or inflammatory response (Hori and Kim 2019; Lee et al. 2022). Notably, the pronounced elevation in the risk of metabolic syndrome (Mellon et al. 2018; Michopoulos, Vester, and Neigh 2016) and autoimmune diseases (O'Donovan et al. 2015; Hsu et al. 2024) both linked to immune dysregulation, among individuals with PTSD further underscores this connection. The analysis of blood biomarkers, including interleukin (IL)‐1β, IL‐6, tumor necrosis factor (TNF‐α), and C‐reactive protein (CRP), which are secreted by peripheral immune cells such as macrophages and lymphocytes, has consistently demonstrated significant elevations in PTSD patients compared to healthy controls (Dalgard et al. 2017; von Känel et al. 2007; Wang et al. 2016, 2019). Additionally, the observed decline in cortisol levels in individuals with PTSD, accompanied by an upsurge in pro‐adrenal hormone‐releasing hormone secretion, exacerbates the inflammatory response (Amsterdam and Sasson 2002; Joëls, Fernandez, and Roozendaal 2011). This implies that the inflammatory response assumes a pivotal role in the pathogenesis and pathophysiology of PTSD (Hori and Kim 2019). Although several theories have been postulated to elucidate how PTSD influences immune function and how the inflammatory response intercedes in the pathogenesis of PTSD (Yehuda, McFarlane, and Shalev 1998; Fonzo et al. 2010), additional empirical research is warranted to establish a causal relationship between these phenomena.

Mendelian randomization (MR) study is currently acknowledged as a highly appealing alternative to traditional randomized controlled trials (RCTs) in the realm of causal inference (Bowden and Holmes 2019). MR study leverages single nucleotide polymorphisms (SNPs) as instrumental variables, which adhere to natural random assignment, remain impervious to external environmental influences, and can be precisely and directly measured. In comparison to conventional RCTs, MR study exhibits a superior capacity to mitigate biases and yield robust causal estimates elucidating the association between exposure and outcome (Tin and Köttgen 2021). Consequently, we conducted the forward and backward two‐sample MR analyses, utilizing publicly available summary‐level data from genome‐wide association studies (GWAS), to investigate the causal relationship between PTSD and immune function.

## 2 Methods

### 2.1 Overall Study Design

**FIGURE 1:**
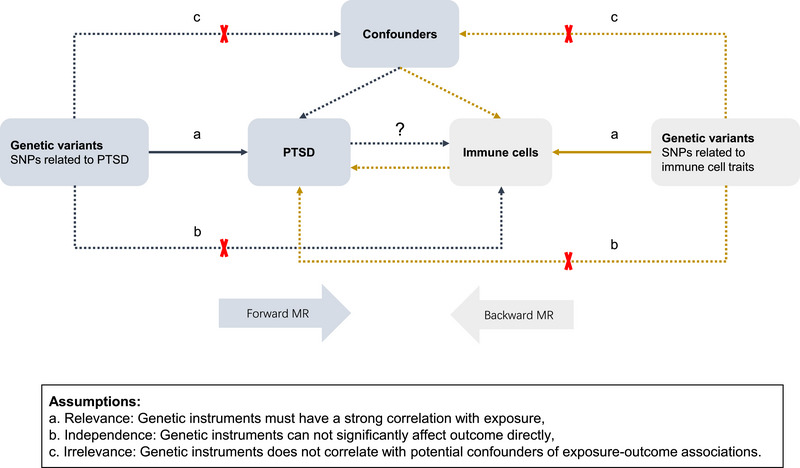
Schematic diagram of a Mendelian randomization study. Forward MR analysis: PTSD as exposure, immune cell traits as outcome. Backward MR analysis: immune cell traits as exposure, PTSD as outcome. The bottom diagram shows the three core assumptions of the MR study. MR, Mendelian randomization; PTSD, post‐traumatic stress disorder; SNP, single nucleotide polymorphism.

The workflow of the bidirectional MR study is depicted in Figure 1. In the forward MR analysis, PTSD was employed as the exposure, and immune cell traits served as the outcome. Conversely, in the backward MR analysis, immune cell traits were used as the exposure, with PTSD as the outcome. Figure 1 also illustrates the satisfaction of the three pivotal assumptions pertaining to the genetic variants employed in the exposure inclusion.

### 2.2 Data Source of PTSD

The PTSD data were derived from a large GWAS meta‐analysis of 43 cohorts, including 174,659 individuals (23,212 cases and 151,447 controls) of European ancestry (Nievergelt et al. 2019). This GWAS identified only two genome‐independent significant loci at the genome‐wide significance level (*p* < 5 × 10^−8^) and found a high genetic correlation between PTSD and immune system disorders such as asthma (*r*_g_ = 0.49, *p* = 0.0002).

### 2.3 Data Source of Immune Cells Traits

GWAS data on immune cell traits were obtained from a cohort of 3757 Sardinians (Orrù et al. 2020). A total of 731 immune features were analyzed in the study, which included B‐cells, conventional dendritic cells (DCs), mature stage T‐cells, monocytes, myeloid cells, TBNK (T‐cells, B‐cells, natural killer cells), and Treg cells. All cell types were also detected and categorized according to the four methods of absolute cell counts (AC), relative cell counts (RC), median fluorescence intensities (MFI), and morphometric parameters (MP). Summary‐level data, adjusted for covariates of sex and age, were publicly available in the GWAS catalog (https://www.ebi.ac.uk/gwas/studies/). The accession numbers were from GCST0001391 to GCST0002121.

### 2.4 Selection of Genetic Variants

For the forward MR analysis, we set the significance level to 5 × 10^−6^ to extract more genetic variants in order to obtain a reliable causal relationship of PTSD on immune cell traits. For the backward MR analysis, we set the significance level to 1 × 10^−5^ to extract genetic variants for each immune cell trait, as has been adopted in a previous study in disentangling bidirectional relationships between immune cells traits and schizophrenia (Wang et al. 2023). The process of selecting independent SNPs involved linkage disequilibrium clumping, employing an *r* ^2^ threshold of < 0.001 within a 10,000 kb distance. Calculations of linkage disequilibrium were predicated on the reference dataset from the European 1000 Genome Project. Furthermore, *F*‐statistics, calculated as beta^2^/se^2^ (Perry et al. 2021), were utilized to assess weak instrumental bias and instrument strength, with a threshold of *F*‐statistics > 10 deemed essential for the subsequent MR analysis.

### 2.5 Statistical Methods

To establish robust causality between the exposure and the outcome in our study, we primarily employed the random‐effects inverse variance weighting (IVW) method, which effectively excludes the effect of heterogeneity in the presence of genetic variants on the outcome (Lin, Deng, and Pan 2021). In addition to IVW (fixed‐effects), we complemented our analysis with the MR‐Egger method, the weighted median method, the weighted modeling method, and the simple model method to fortify the causality assessment of the results.

To assess the presence of horizontal pleiotropy in the genetic variants, we employed the MR‐Egger intercept method and the MR‐polytomous residuals and outliers (MR‐PRESSO) method. Horizontal pleiotropy was considered to be present if the *p* value < 0.05, and the MR‐PRESSO method allowed us to identify and exclude outliers that had a substantial impact on the results. Heterogeneity was tested using Cochran's *Q*‐test, and heterogeneity was considered to exist when the *p* value < 0.05. The influence of heterogeneity on causal estimation was further assessed using the leave‐one‐out method, where results were deemed robust if the remaining instrumental variables consistently pointed in the same direction after excluding a particular variable.

Our MR analyses were conducted using the TwoSampleMR and MR‐PRESSO packages within the R language (version 4.2.0, www.r‐project.org). All *p* values in this study are two‐tailed. The false discovery rate (FDR) correction was applied to account for multiple tests across different panels within each category.

## 3 Results

### 3.1 Forward MR Analysis: Causal Effects of PTSD on Immune Cell Traits

**FIGURE 2:**
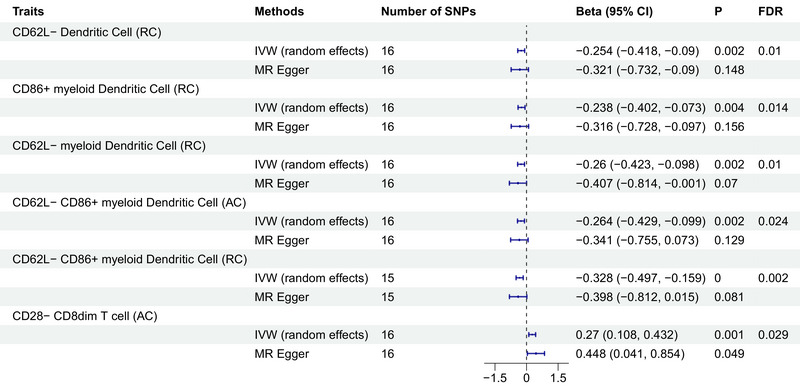
Causal effects of PTSD on immune cell traits. Forest plots showed the causal associations of PTSD on immune cell traits. AC, absolutely counts; CI, confidence interval; FDR, false discovery rate; IVW, inverse variance weighting; MR, Mendelian randomization; PTSD, post‐traumatic stress disorder; RC, relative counts; SNP, single nucleotide polymorphism.

Our analysis revealed that PTSD exerted significant causal effects on six specific immune cell traits. Five of these immune cell traits, all categorized under DCs, exhibited a negative causal association with PTSD, whereas immune cell traits within the T‐cell category displayed a positive causal association with PTSD. Notably, employing the random‐effects IVW method as the gold standard, we identified the following outcomes: PTSD was found to reduce the levels of CD62L− DC %DC (beta = −0.254, *p* = 0.002, FDR = 0.01), CD86+ myeloid DC %DC (beta = −0.238, *p* = 0.004, FDR = 0.014), CD62L− myeloid DC %DC (beta = −0.26, *p* = 0.002, FDR = 0.01), CD62L− CD86+ myeloid DC absolute count (beta = −0.264, *p* = 0.002, FDR = 0.024), and CD62L− CD86+ myeloid DC %DC (beta = −0.328, *p* < 0.001, FDR = 0.002). In contrast, PTSD was observed to increase the level of CD28− CD8dim T cell absolute count (beta = 0.27, *p* = 0.001, FDR = 0.029) (Figure 2, Tables S1 and S2). Importantly, both the MR‐Egger's intercept and the MR‐PRESSO's global test were utilized to assess the presence of pleiotropy, and no significant evidence of pleiotropy was detected (Table 1). Furthermore, scatter plots and leave‐one‐out analysis consistently demonstrated the direction of causality, thereby affirming the stability of the obtained results (Figures S1 and S2).

**TABLE 1:** Sensitive analysis of post‐traumatic stress disorder (PTSD) on immune cell traits.

			MR‐Egger regression		MR‐PRESSO		Heterogeneity analyses		
Exposure	Outcome	Number of SNPs	Intercept	*p*		Global test *p*	Method	*Q*	*Q*‐*p*val
PTSD	CD62L− CD86+ myeloid DC %DC	15	0.009	0.723	9.65	0.945	MR‐Egger	7.726	0.903
							IVW	7.857	0.929
PTSD	CD62L− DC %DC	16	0.008	0.733	10.824	0.925	MR‐Egger	6.503	0.970
							IVW	6.623	0.980
PTSD	CD62L− myeloid DC %DC	16	0.019	0.453	9.286	0.966	MR‐Egger	7.147	0.953
							IVW	7.739	0.956
PTSD	CD86+ myeloid DC %DC	16	0.009	0.704	4.999	0.999	MR‐Egger	5.988	0.980
							IVW	6.137	0.987
PTSD	CD62L− CD86+ myeloid DC AC	16	0.010	0.703	10.265	0.942	MR‐Egger	9.220	0.866
							IVW	9.371	0.897
PTSD	CD28− CD8dim AC	16	−0.022	0.363	20.045	0.435	MR‐Egger	11.967	0.682
							IVW	12.846	0.684

Abbreviations: AC, absolutely counts; DC, dendritic cell; IVW, inverse variance weighting; MR, Mendelian randomization; MR‐PRESSO, MR‐polytomous residuals and outlier; SNP, single nucleotide polymorphism.

### 3.2 Backward MR Analysis: Causal Effects of Immune Cell Traits on PTSD

**FIGURE 3:**
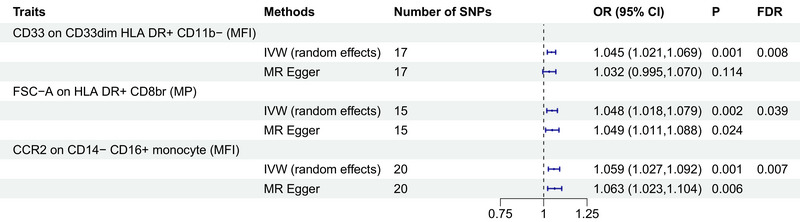
Causal effects of immune cell traits on PTSD. Forest plots showed the causal associations of immune cell traits on PTSD. CI, confidence interval; FDR, false discovery rate; IVW, inverse variance weighting; MFI, median fluorescence intensities; MP, morphometric parameters; MR, Mendelian randomization; PTSD, post‐traumatic stress disorder.

We have identified three immune cell traits associated with an increased risk of developing PTSD. Notably, the odds ratio (OR) for CD33 on CD33dim HLA DR+ CD11b− in relation to PTSD risk was found to be 1.045 (95% CI = 1.021–1.069, *p* = 0.001, FDR = 0.008). Additionally, the OR for FSC‐A on HLA DR+ CD8br in relation to PTSD risk was calculated at 1.048 (95% CI = 1.018–1.079, *p* = 0.002, FDR = 0.039), and for CCR2 on CD14− CD16+ monocyte, the OR was determined to be 1.059 (95% CI = 1.027–1.092, *p* = 0.001, FDR = 0.008) (Figure 3, Tables S3 and S4). Both MR‐Egger's intercept and MR‐PRESSO's global test excluded any significant pleiotropic effects (Table 2). Moreover, the results were substantiated by scatter plots and leave‐one‐out analysis, demonstrating the robustness of our findings (Figure S3).

**TABLE 2:** Sensitive analysis of immune cell traits on post‐traumatic stress disorder (PTSD).

			MR‐Egger regression		MR‐PRESSO		Heterogeneity analyses		
Exposure	Outcome	Number of SNPs	Intercept	*p*		Global test *p*	Method	*Q*	*Q*‐*p*val
CCR2 on CD14− CD16+ monocyte	PTSD	20	−0.003	0.768	30.505	0.308	MR‐Egger	21.783	0.242
							IVW	21.892	0.290
CD33 on CD33dim HLA DR+ CD11b−	PTSD	17	0.007	0.398	17.227	0.698	MR‐Egger	8.214	0.915
							IVW	8.970	0.915
FSC‐A on HLA DR+ CD8br	PTSD	15	0.000	0.964	20.868	0.423	MR‐Egger	14.197	0.360
							IVW	14.199	0.435

Abbreviations: DC, dendritic cell; IVW, inverse variance weighting; MR, Mendelian randomization; MR‐PRESSO, MR‐polytomous residuals and outlier; SNP, single nucleotide polymorphism.

## 4 Discussion

To the best of our knowledge, this is the first MR study to explore the causal relationship between multiple immune cell traits and PTSD. Among 731 immune cell traits, we found that PTSD had a significant causal effect on six cell traits, including a negative causal effect on five cell traits, all of which are DCs, and a positive causal effect on T cells. In addition, three immune cell traits showed significant positive causal effects on PTSD, suggesting that they increase the risk of developing PTSD.

DCs are the most potent specialized antigen‐presenting cells and play an important role in the initiation and regulation of innate and adaptive immune responses (Waisman et al. 2017). Alterations in blood DCs are common in autoimmune diseases (Brandum et al. 2021; Ludewig et al. 2001), cancer (Lee and Radford 2019), and central nervous system disorders (Mrdjen et al. 2018; Sie and Korn 2017), and studies on DCs and neuropsychiatric disorders have found reduced blood DCs levels in AD patients (Ciaramella et al. 2016). The causes of reduced blood DCs are complex and may be related to altered DCs viability, mobilization, or impaired differentiation of them from progenitor cells, and there is also the possibility of blood DCs‐specific metastasis to diseased tissues (Karman 2004). Although there is no direct research evidence, given that PTSD may be a subtype of depression, and in combination with the study's finding that low DCs levels are associated with increased depression in AD patients (Ciaramella et al. 2016), it is not difficult to understand the plausibility of a causal relationship between PTSD on lower levels of DCs.

Higher levels of IL‐1β, IL‐6, and TNF‐α have been found in the plasma of PTSD patients compared to normal controls (von Känel et al. 2007; Spivak et al. 1997; Maes et al. 1999), and the altered levels of these cytokines suggest that peripheral blood monocytes from PTSD patients have been pre‐activated in the body (Gola et al. 2013). The results we found on monocytes and the risk of developing PTSD provide further evidence for the relationship between cytokines and PTSD and strongly suggest that chronic low‐level inflammation may be a potential mechanism for the development of PTSD (von Känel et al. 2007; Gola et al. 2013). Notably, we identified a complex relationship between T cells and PTSD that appears to be distinct from other immune cell traits. Previous studies have identified elevated T‐lymphocyte counts in patients with PTSD (Gola et al. 2013; Aiello et al. 2016) and suggested that this is related to a decrease in their cortisol levels (Yehuda, McFarlane, and Shalev 1998; Yehuda et al. 1995) and consequently a pro‐inflammatory cytokine response (Rohleder et al. 2003). Our study further establishes a reciprocal causal relationship, although the two results derive from different immune cell traits. This strongly suggests that T cells perhaps play a more far‐reaching role in the development of PTSD than other immune cells and could perhaps be the most promising target in the use of immunotherapeutic strategies to prevent and treat PTSD.

Although our MR study leveraged a large‐scale GWAS cohort and the causal estimates were diligently obtained through a rigorous screening of instrumental variables, certain limitations warrant consideration. First, given the current lack of population‐specific GWAS data, it is premature to generalize these results to the broader European population, because it is important to acknowledge that the study cohorts of PTSD were exclusively sourced from European populations. However, the immune cell signature cohort comes from a Sardinian population in Italy, which, although geographically part of Europe, has an ethnicity and genome that, strictly speaking, have deeper historical roots in North Africa. A degree of caution is also warranted when extending our findings to other ethnic groups, as genetic and environmental factors may vary across diverse populations. Additionally, although the study aimed to explore causality, the influence of gender on the observed relationships cannot be entirely excluded. However, conducting an in‐depth gender‐specific analysis posed challenges because publicly available immune cell trait data are typically adjusted for covariates like sex and age, potentially obscuring a nuanced examination of gender‐related effects. Due to the constraints of the GWAS data source, we were unable to stratify the MR study by gender. Consequently, the current results reflect only the causal relationship between the overall PTSD population and immune cell characteristics. It is important to emphasize that this does not imply that PTSD is unrelated to gender. Rather, it is widely accepted that the higher prevalence of PTSD in females is well‐documented (Christiansen and Berke 2020) and is by no means accidental. A further limitation is that PTSD is a broad diagnosis, and whether the same causal relationship exists under its subdiagnoses or sub‐symptoms with immune cell characteristics needs to be explored in depth in the future.

To conclude, our bidirectional MR study unearthed a causal relationship between immune cell traits and PTSD, shedding light on the intricate interplay between the immune function and PTSD. Notably, the identification of a bidirectional causal relationship between T cells and PTSD opens new avenues for considering innovative approaches to the prevention and early intervention in PTSD. This study contributes to a deeper understanding of the underlying mechanisms and offers valuable insights into the potential management of PTSD.

## Author Contributions

J.W. and J.D. designed the study and wrote the protocol. Y.S. and X.D. managed the literature searches and analyses. X.D. and J.D. undertook the statistical analysis. J.W. and Y.S. wrote the first draft of the manuscript. All authors contributed to and have approved the final manuscript.

## Ethics Statement

Ethical approval was obtained for all cohorts in the included original studies with publicly available GWAS data. All data are publicly available and are approved by the institutional review committees in their respective studies. Therefore, no further sanction was required.

## Consent

Consent to participate was obtained for all cohorts in the included original studies with publicly available GWAS pooled data.

## Conflicts of Interest

The authors declare no conflicts of interest.

### Peer Review

The peer review history for this article is available at https://publons.com/publon/10.1002/brb3.70073.

## Supporting information



Additional supporting information can be found online in the Supporting Information section.

Additional supporting information can be found online in the Supporting Information section.

Additional supporting information can be found online in the Supporting Information section.

Additional supporting information can be found online in the Supporting Information section.

Additional supporting information can be found online in the Supporting Information section.

Additional supporting information can be found online in the Supporting Information section.

Additional supporting information can be found online in the Supporting Information section.

Additional supporting information can be found online in the Supporting Information section.

## Data Availability

The original contributions presented in the study are included in the article, and further inquiries can be directed to the corresponding authors.
