# Supplementary figures and images for: Causal Relationship Between Post‐Traumatic Stress Disorder and Immune Cell Traits: A Mendelian Randomization Study
Source: Brain Behav. 2024 Sep 30;14(10):e70073. doi: 10.1002/brb3.70073 (PMC11443039; doi:10.1002/brb3.70073)

Supplementary Figure 1

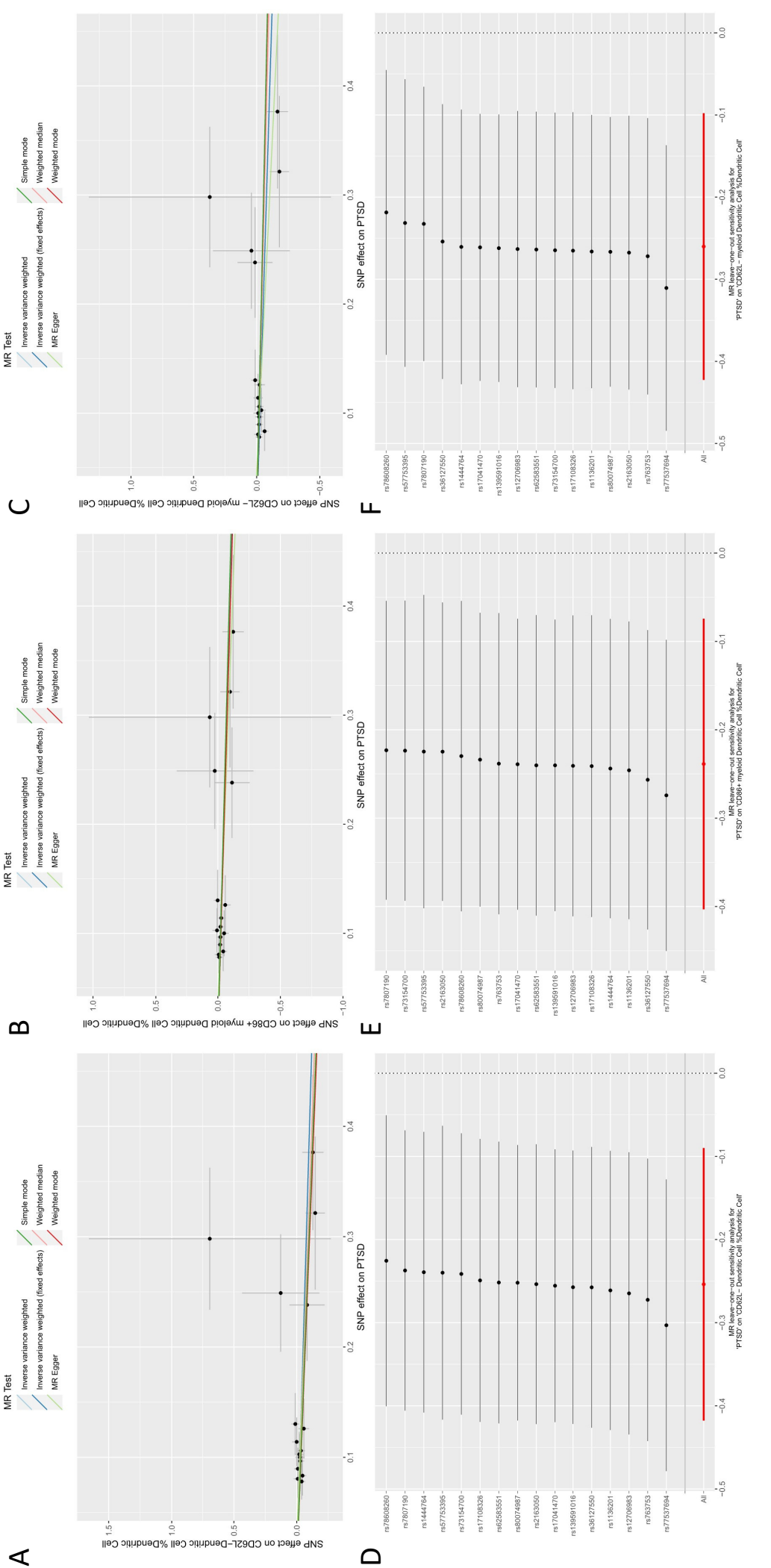

Supplement: Supplementary file 1 — Additional supporting information can be found online in the Supporting Information section. [file BRB3-14-e70073-s003.pdf]

Supplementary Figure 2

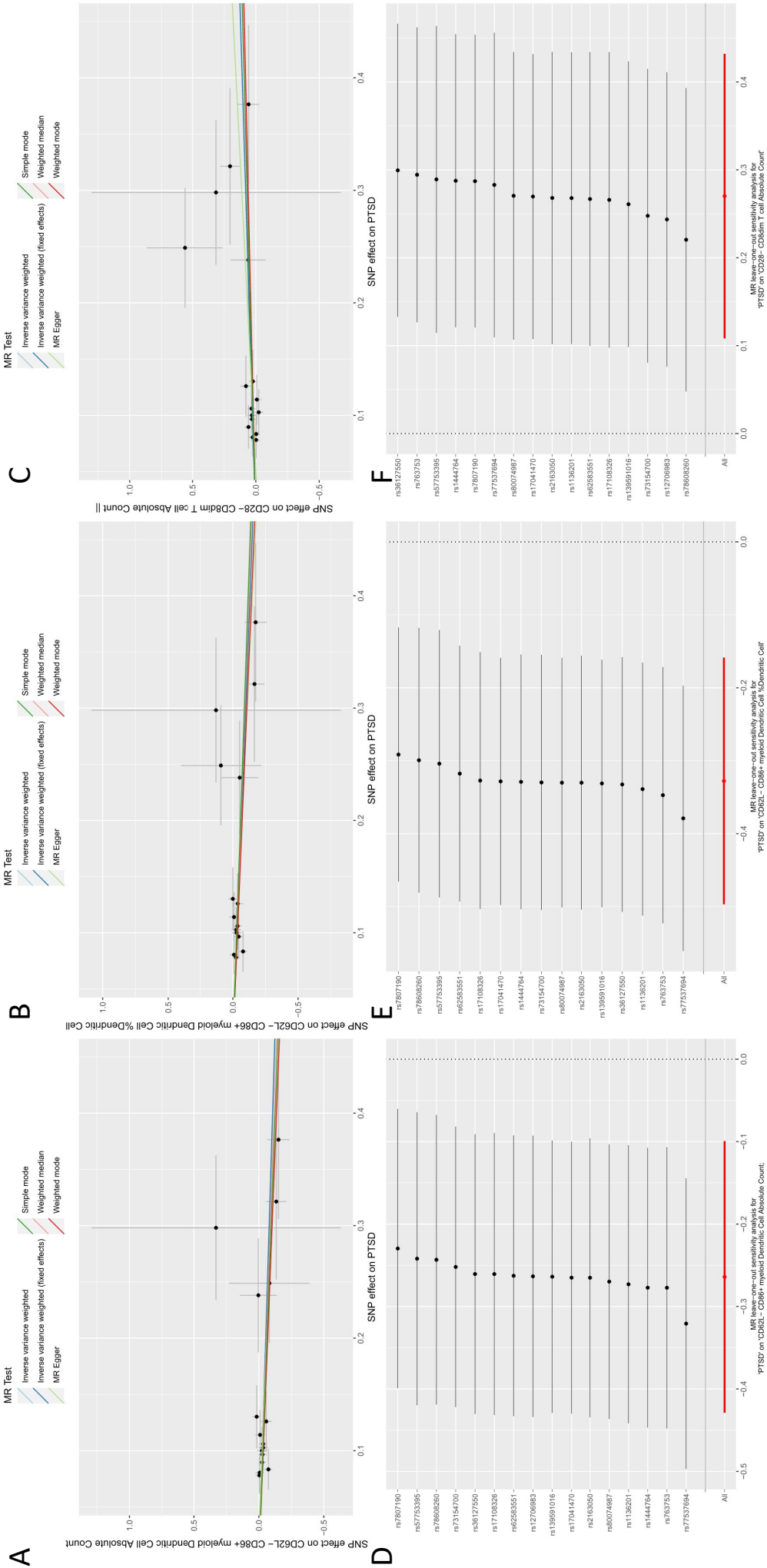

Supplement: Supplementary file 2 — Additional supporting information can be found online in the Supporting Information section. [file BRB3-14-e70073-s008.pdf]

Supplementary Figure 3

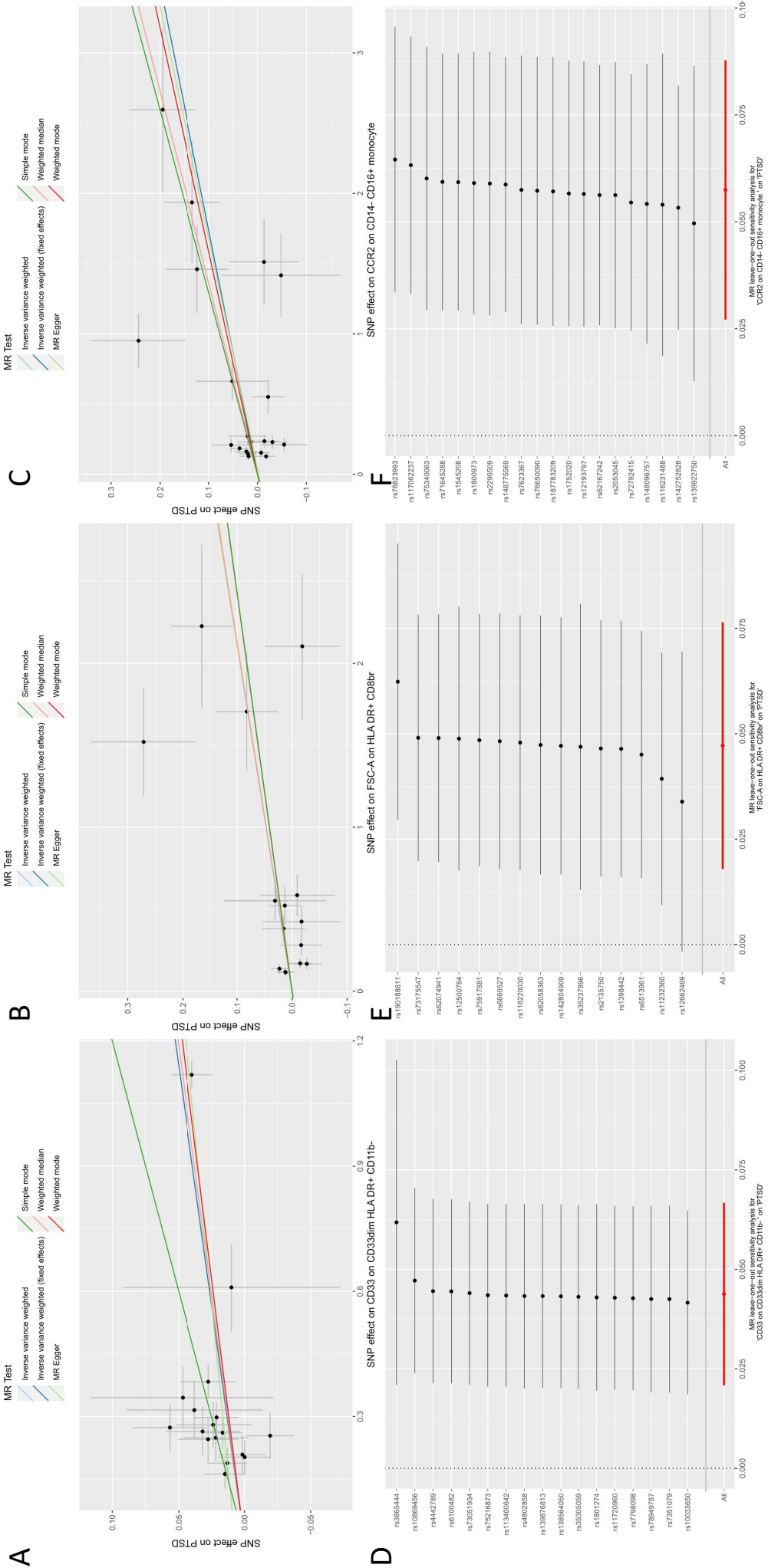

Supplement: Supplementary file 3 — Additional supporting information can be found online in the Supporting Information section. [file BRB3-14-e70073-s007.pdf]
