# Supplementary material for: Causal Relationship Between Post‐Traumatic Stress Disorder and Immune Cell Traits: A Mendelian Randomization Study
Source: Brain Behav. 2024 Sep 30;14(10):e70073. doi: 10.1002/brb3.70073 (PMC11443039; doi:10.1002/brb3.70073)
Supplement: Supplementary file 4 — Additional supporting information can be found online in the Supporting Information section. [file BRB3-14-e70073-s005.docx]

**Supplementary Figures Legends**

**Supplementary Figure 1.** Visualization of the causal effect of posttraumatic stress disorder (PTSD) on immune cell traits (Part 1). Scatter plots demonstrate the direction of the causal effect of PTSD on immune cell traits, and Leave-one-out analysis suggests the stability of the causal effect. (A, D) The causal effect of PTSD on CD62L- Dendritic Cell. (B, E) The causal effect of PTSD on CD86+ Myeloid Dendritic Cell. (C, F). The causal effect of PTSD on CD62L- Myeloid Dendritic Cell. PTSD: posttraumatic stress disorder; SNP: single nucleotide polymorphism.

**Supplementary Figure 2.** Visualization of the causal effect of PTSD on immune cell traits (Part 2). Scatter plots demonstrate the direction of the causal effect of PTSD on immune cell traits, and Leave-one-out analysis suggests the stability of the causal effect. (A, D) The causal effect of PTSD on CD62L- CD86+ Myeloid Dendritic Cell Absolute Count. (B, E) The causal effect of PTSD on CD62L- CD86+ Myeloid DC. (C, F). The causal effect of PTSD on CD28- CD8dim T cell Absolute Count. PTSD: posttraumatic stress disorder; SNP: single nucleotide polymorphism.

**Supplementary Figure 3.** Visualization of the causal effect of immune cell traits on PTSD. Scatter plots demonstrate the direction of the causal effect of PTSD on immune cell traits, and Leave-one-out analysis suggests the stability of the causal effect. (A, D) The causal effect of CD33 on CD33dim HLA DR+ CD11b- on PTSD. (B, E) The causal effect of FSC-A on HLA DR+ CD8br on PTSD. (C, F). The causal effect of CCR2 on CD14- CD16+ monocyte on PTSD. PTSD: posttraumatic stress disorder; SNP: single nucleotide polymorphism.
